# No health without access: using a retrospective cohort to model a care continuum for people released from prison at an urban, safety net health system

**DOI:** 10.1186/s40352-023-00248-3

**Published:** 2023-11-18

**Authors:** Michael Frank, Ryan Loh, Rachel Everhart, Hermione Hurley, Rebecca Hanratty

**Affiliations:** 1 Denver Health and Hospital Authority, Denver, CO USA; 2 Department of Medicine, University of Colorado School of Medicine, Aurora, CO USA; 3 Department of Psychiatry, University of Colorado School of Medicine, Aurora, CO USA

**Keywords:** Prisons, Prisoners, Continuity of patient care, Community health services, Health services accessibility, Outpatient, Medicaid, Mental disorders, United States

## Abstract

**Background:**

Release from prison is characterized by discontinuity of healthcare services and results in poor health outcomes, including an increase in mortality. Institutions capable of addressing this gap in care seldom collaborate in comprehensive, data-driven transition of care planning. This study harnesses information from a data exchange between correctional facilities and community-based healthcare agencies in Colorado to model a care continuum after release from prison.

**Methods:**

We merged records from Denver Health (DH), an urban safety-net healthcare system, and the Colorado Department of Corrections (CDOC), for people released from January 1 to June 30, 2021. The study population was either (a) released to the Denver metro area (Denver and its five neighboring counties), or (b) assigned to the DH Regional Accountable Entity, or (c) assigned to the DH medical home based on Colorado Department of Healthcare Policy and Financing attribution methods. Outcomes explored were outpatient, acute care, and inpatient utilization in the first 180 days after release. We used Pearson’s chi-squared tests or Fisher exact for univariate comparisons and logistic regression for multivariable analysis.

**Results:**

The care continuum describes the healthcare utilization at DH by people released from CDOC. From January 1, 2021, to June 30, 2021, 3242 people were released from CDOC and 2848 were included in the data exchange. 905 individuals of the 2848 were released to the Denver metro area or attributed to DH. In the study population of 905, 78.1% had a chronic medical or psychological condition. Within 180 days of release, 31.1% utilized any health service, 24.5% utilized at least one outpatient service, and 17.1% utilized outpatient services two or more times. 10.1% utilized outpatient services within the first 30 days of release.

**Conclusions:**

This care continuum highlights drop offs in accessing healthcare. It can be used by governmental, correctional, community-based, and healthcare agencies to design and evaluate interventions aimed at improving the health of a population at considerable risk for poor health outcomes and death.

**Supplementary Information:**

The online version contains supplementary material available at 10.1186/s40352-023-00248-3.

## Background

Over one-half million people are released from prison in the US per year, and 95% of people incarcerated in prison will eventually be released (Carson, 2021; Hughes, 2004). People who are incarcerated have higher medical, mental health, and substance use disorder treatment needs than the general population (Binswanger et al., 2009; California Health Policy Strategies, 2020; Davis et al., 2011). Rather than a seamless transition of health services, discontinuity of care is the norm (Puglisi et al., 2017).

Accessing healthcare after incarceration requires overcoming numerable barriers. In qualitative studies, individuals being released from prison describe housing instability, low income, inadequate transportation, difficulty with timely access to primary care, burdens imposed by community supervision (i.e., parole), healthcare-induced trauma, and coping with substantial stress, anxiety, and fear experienced when getting out of prison (Binswanger et al., 2011; Elumn et al., 2021). In addition, people must contend with criminal justice history discrimination, low health literacy, and a lack of health insurance coverage (Hadden et al., 2018; Redmond et al., 2020; Winkelman et al., 2017).

Given these barriers accessing care, people released from prison have higher rates of emergency care utilization and hospitalizations and lower rates of outpatient care utilization than the general population (Frank et al., 2014; Wang et al., 2013). In the first two weeks after release from prison, the rates of death and overdose are up to 12 and 129 times that of the general population, respectively (Binswanger et al., 2007). Reentry is characterized by additional negative health outcomes, including untreated substance use and psychiatric disorders, traumatic injuries, suicide, cardiovascular disease, and cancer (Binswanger et al., 2007; Borschmann et al., 2017; CDC, 2007; Hagan et al., 2018; Howell et al., 2016; Puglisi et al., 2020; Young et al., 2018). Discontinuity of healthcare results in devastating outcomes for a population already laden with a disproportionate burden of health problems.

Correctional and community healthcare seldom collaborate in transitions of care from prison, leaving the two systems essentially siloed (Butler, 2014; Davis, 2015; Marks & Turner, 2014; Commonwealth Fund, 2020; Puglisi et al., 2022). Although there is a federal mandate to provide basic medical care inside prisons, there are no legal mandates or incentives for conducting pre-release planning to ensure continuity of healthcare services (Puglisi et al., 2022). In the literature, we are only able to identify a few examples of data sharing, which aim to improve transitions of care, between correctional and community health systems (Jannetta et al., 2018; Hinchman et al., 2018; Milgram A, 2018; Trestman & Aseltine, 2014). Nevertheless, both community and correctional providers support the proliferation of health information exchanges as a strategy that can improve access to care for people being released from prison (NCCHC Governance Board, 2021; Divakaran et al., 2022).

Improving the health of people getting out of prison requires, not only the aforementioned data sharing and collaboration between correctional and community health systems, but also the development and consistent use of tools that monitor access to healthcare in the community. One crucial tool to improve the health of a population is found in the care continuum model. Continuums of care look at an entire population that shares a common feature. They can identify major drop-offs in accessing care, reveal unexpected patterns of healthcare utilization, inform future interventions, and provide data for the evaluation of those interventions. Care continuums have been developed for people living with HIV, hepatitis C, and opioid use disorder (Gardner et al., 2011; Kamis et al., 2022a, b; Prieto et al., 2019). Subsequently, they are incorporated into a health system’s routine tracking and evaluation to improve health outcomes. In this paper we show how the continuum of care model can be applied to the population released from prison.

In 2015 a multilateral partnership in the state of Colorado was launched. With the passing of the Affordable Care Act and expansion of Medicaid in Colorado, it became evident that many people being released from incarceration would be eligible for Medicaid (Bandara et al., 2015). With the goal of improving the transition of care from prison to the community, the Colorado Department of Corrections (CDOC) began sharing rosters of individuals being released from prison with the Colorado Department of Health Care Policy and Financing (HCPF) and, in turn, with Colorado’s Regional Accountable Entities (RAEs) which are responsible for care management in the community setting. Each RAE in Colorado is contractually tasked and monitored for outcomes that demonstrate Medicaid members have access to and utilize primary care and behavioral health services (Bontrager et al., 2018). The Denver Health RAE is the health system analyzed in this study.

By using the validated data from this agreement, this study models a novel care continuum for people released from CDOC to the US metropolitan area served by DH, which is one of the largest providers of care for those insured by Medicaid. Using the care continuum, we can assess the care gaps faced by individuals in their transition of care from prison to the community. Medical information provided by CDOC in this agreement includes markers of medical and psychological disease severity. These markers enable us to examine the differences in accessing care among those with and without medical and psychological problems. Our aims are (1) to create and analyze a continuum of healthcare access and utilization for people released from prison and (2) to determine if medical and psychological disease categorization by CDOC is associated with healthcare utilization after release from prison. This study will inform how correctional-community-governmental partnerships can use data sharing and a care continuum model to identify care gaps, direct resources, and evaluate programming to improve the health of people released from prison.

## Methods

### Study design and setting

This was a retrospective cohort study of individuals who were released from CDOC from January 1, 2021, to June 30, 2021, and the healthcare they received in the first six months after release from prison at DH, an integrated safety-net healthcare system in Denver, Colorado. CDOC is the state prison system in Colorado. It comprises 23 private and state administered prisons which incarcerate nearly 20,000 unique individuals annually (Colorado Department of Corrections, 2023). CDOC also provides community corrections supervision to over 10,000 community-dwelling individuals on parole annually. DH services include a 500-bed acute care hospital, a level 1 adult trauma center, 11 federally qualified community health centers serving 180,000 patients in 2021, a public health clinic, a community detoxification unit, ambulance service, an outpatient substance treatment program, and outpatient psychiatric services (Denver Health, 2023). DH serves about a quarter of the Denver population and is the largest healthcare provider in Colorado to people with Medicaid or no insurance. This study was approved by the Colorado Multiple Institutional Review Board.

### Data sources

A data sharing agreement between CDOC and HCPF provides release information to RAEs, one of which is DH (HCPF, 2023). The data is a roster of individuals released from CDOC during the study period. The release rosters only included individuals who were Medicaid-eligible and who signed a release of information allowing their information to be used to improve transitions of care. Elements included in the release roster are full name, date of birth, release date, release address, Medicaid ID number, and medical and psychological diagnostic categories.

For administrative purposes CDOC assigns a medical (M code) and psychological (P code) treatment need category to each incarcerated person. Neither category represents substance use conditions. These codes designate medical and psychological treatment needs on a scale from one to five with increasing numbers indicating greater treatment needs (See Appendix). We refer to these categories as M + or M- and P + or P- to denote the presence or absence of a chronic medical or psychological condition. Specifically, M + represents anything from the early stages of a chronic condition to medical problems requiring specialty care and end stage chronic conditions. M- represents no medical needs. P + represents a broad spectrum of individuals, including those taking any type of mental health medication, having a serious mental illness, or being a current danger to oneself or others. Lastly, P- represents individuals with no mental health treatment needs or no history of recent or active psychological problems. For clarity, these groups will be referred to as the following: M+, chronic medical conditions; M-, no chronic medical conditions; P+, chronic psychological conditions; P-, no chronic psychological conditions.

### Study population

A total of 3242 individuals were released from CDOC in the study timeframe, 2848 of which were included in the release roster. CDOC releases individuals to jurisdictions across the state of Colorado, often geographically distant from DH. To focus the analysis on individuals who would be more likely to receive care at DH (attributed to DH), the release roster was filtered to include the 905 individuals who either (a) were released to the Denver metro area (Denver and its five neighboring counties), or (b) were assigned to the DH RAE, or (c) were assigned to DH as the patient’s medical home by HCPF based on attribution methods (HCPF, 2023). Because of the limited demographic information available in the release roster, a deterministic matching algorithm utilized the patients first name, last name, date of birth, and Medicaid number to identify patients in the DH medical record. Patients that could not be matched to any record in the DH medical record were not included in the study. Full demographic variables were only available for matched patients using the DH electronic health record.

### Outcome measures and statistical analyses

All encounters that included a healthcare provider were included, including telehealth encounters. These encounters were categorized as acute care (emergency and urgent care), outpatient care (office visits in primary care, specialty care, behavioral health, and substance use treatment), or hospitalizations. This grouping of acute care compared to outpatient was used because establishing and engaging with primary care, outpatient behavioral health, and substance use care improves health outcomes for people released from prison (Divakaran et al., 2022). It also provides the structure for modeling a care continuum for our study cohort. A six-month timeframe was selected because the risk of overdose, hospitalization, and death are highest in the first two weeks after release from prison and persist for at least 12 weeks (Binswanger et al., 2007; Wang et al., 2013).

Mortality data were provided by the Colorado Department of Public Health and Environment (CDPHE). DH transmits patient data to CDPHE, records are linked using multiple identifiers (first name, last name, date of birth, and social security number) in a deterministic matching algorithm, and outcomes are securely and electronically returned to the hospital system. The algorithm required matching on three of the four identifiers. State death data include date and cause of death for all residents. Out-of-state deaths are captured by the state through interstate data exchange agreements.

Our analysis began by comparing sociodemographic characteristics between the M + or P + group and the M- and P- group using chi-square tests in order to characterize differences between patients with and without chronic conditions. We then compared the unadjusted difference in healthcare utilization outcomes between the M + or P + group and the M- and P- group using chi-square tests. The chi-square test was chosen given our data represented counts and conformed with the model assumptions that all expected frequencies are greater than zero and no more than 20% of expected frequencies are less than five. Demographic and clinical variables were then included as covariates in multivariable generalized linear models using a logit link to identify predictors of healthcare utilization within six months (180 days) of release. Data cleaning (Python.org, v.3.7) and statistical analyses were performed (SAS enterprise guide, v. 7.1, Cary, NC). Raw anonymized data for n = 905 individuals included in the final data is available.

## Results

From January 1, 2021, to June 30, 2021, 3242 people were released from CDOC. 2848 unique persons were included on the release roster. The inclusion criteria for this study identified 905 individuals who were released to the Denver metro area or attributed to DH and were able to be identified by the matching algorithm and available DH and CDOC data sources. Within the study population, mean age at release was 37.8 years (+/- 9.98 years) and the majority (n = 784, 86.6%) were male (Table 1). The majority were White individuals (n = 414, 45.7%) and the largest ethnic group was non-Hispanic (n = 420, 46.4%), but a large proportion of individuals had missing, declined, or unknown race (n = 325, 35.9%) or Hispanic ethnicity (n = 257, 28.4%) information. Over one-third (n = 344, 38.0%) of the individuals were identified as smokers. Two-thirds (n = 611, 67.5%) were designated M+, and nearly half (n = 415, 45.9%) were designated P+. The majority (n = 707, 78.1%) were designated M + or P+.

**Table 1:** Sociodemographic characteristics of patients by presence of CDOC chronic condition codes

	Total N (%)	M- and P- (No Chronic Condition) N (%)	M + or P+ (Chronic Condition) N (%)
Total	905	198	707
Age at release (mean) ***	37.8 years	33.9 years	38.9 years
18–25	82 (9.06)	28 (14.14)	54 (7.64)
26–35	331 (36.57)	93 (46.97)	238 (33.66)
36–45	284 (31.38)	57 (28.79)	227 (32.11)
46–55	152 (16.80)	17 (8.59)	135 (19.09)
56+	56 (6.19)	3 (1.52)	53 (7.50)
Sex *** †			
Male	784 (86.63)	192 (96.97)	592 (83.73)
Race * ‡			
American Indian or Alaska Native	12 (1.33)	4 (2.02)	8 (1.13)
Asian	5 (0.55)	2 (1.01)	3 (0.42)
Black or African American	148 (16.35)	25 (12.63)	123 (17.40)
Native Hawaiian	1 (0.11)	0 (0.00)	1 (0.14)
White or Caucasian	414 (45.75)	76 (38.38)	338 (47.81)
Ethnicity ***			
Hispanic	228 (25.19)	57 (28.79)	171 (24.19)
Non-Hispanic	420 (46.41)	66 (33.33)	354 (50.07)
Smoking Status ***			
Smoker (current every/someday smoker)	344 (38.01)	57 (28.79)	287 (40.59)
**Medical Codes**			
M-	294 (32.49)	198 (100.00)	96 (13.58)
M+	611 (67.51)	0 (0.00)	611 (86.42)
**Psychological Codes**			
P-	490 (54.14)	198 (100.00)	292 (41.30)
P+	415 (45.86)	0 (0.00)	415 (58.70)

M+: Medical code 2 and higher

M-: Medical code 1

P+: Psychological code 3 and higher

P-: Psychological code 2 and less

†: N = 1 “Unknown” was not considered

‡: Due to failure to reach Cochran’s standard, groups were consolidated to White or Caucasian vs. Non-White or Caucasian.

*: p < .05

**: p < .01

***: p < .001

**Fig. 1:**
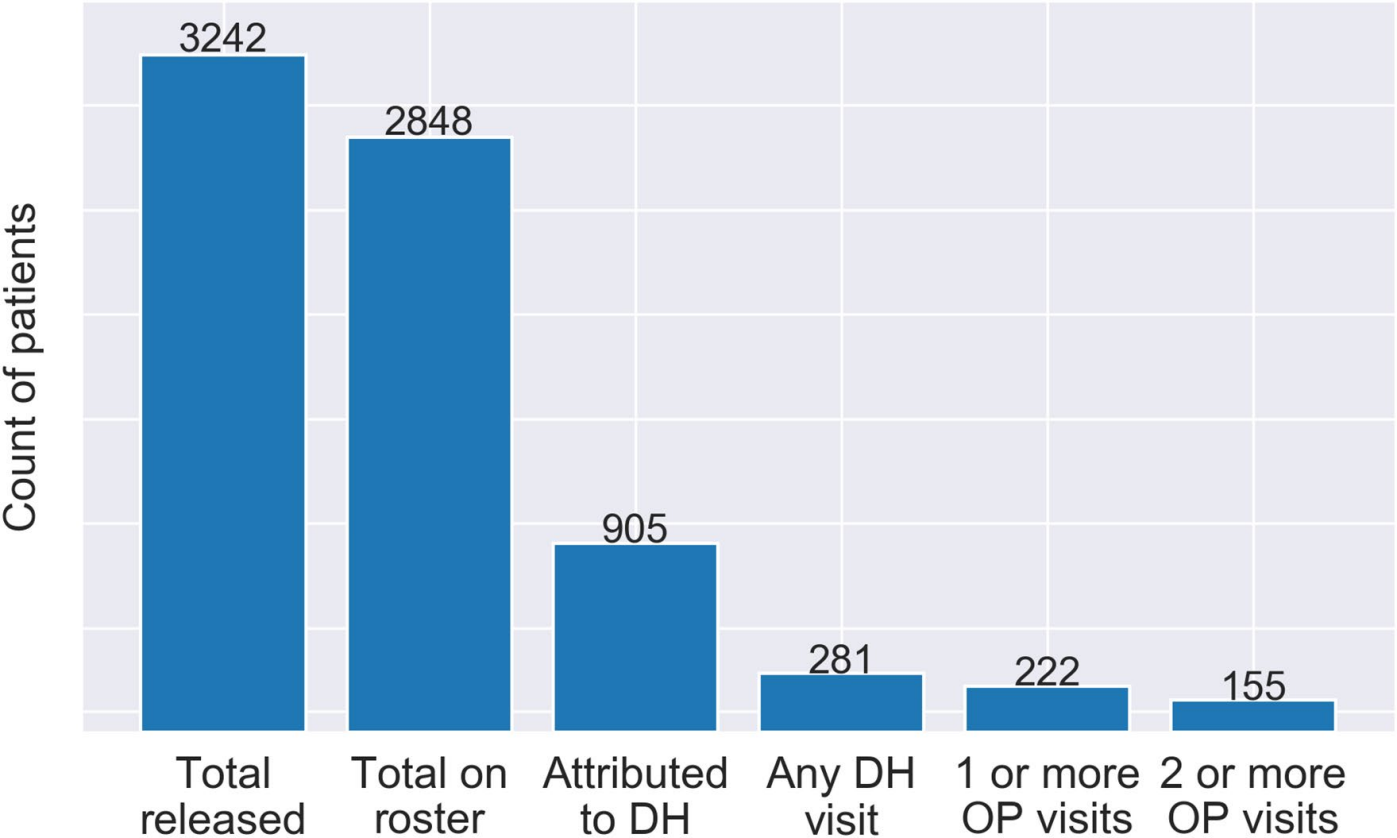
Continuum of healthcare utilization after release from prison

The care continuum (Fig. 1) established that 281 (31.1%) of the 905 individuals utilized any service at DH within 180 days of release. At least one outpatient service was utilized by 222 (24.5%) individuals and 155 (17.1%) of individuals utilized outpatient services two or more times. Outpatient services were utilized within the first 30 days of release by 91 (10.1%) individuals.

There was a statistical difference in utilization between the large subset of individuals in the M + or P + group as compared to the smaller subset of individuals in the M- and P- group (Table 2). An outpatient service within the first 30 days of release was utilized by 12.2% of the M + or P + group as compared to 2.5% of individuals in the M- and P- group (p < .001). At least one outpatient service within 180 days of release was utilized by 27.3% of the M + or P + group as compared to 14.6% of the M- and P- group (p < .001), and two or more outpatient services within 180 days of release were utilized by 19.2% of the M + or P + group versus 9.6% of the individuals in the M- or P- group (p = .002). Utilization of acute care services was also significantly different between the two groups. Acute care utilization was higher for the M + or P + group at 8.2% within 30 days of release and 20.5% within 180 days of release (p = .019), compared to 3.0% within 30 days of release and 13.1% within 180 days of release in the M- and P- group (p = .025).

**Table 2:** Bivariate association of medical and psychological codes with healthcare utilization

	Total N (%)	M- and P- (No Chronic Condition) N (%)	M + or P+ (Chronic Condition) N (%)
**Acute care encounter**			
within 30 days of release *	64 (7.07)	6 (3.03)	58 (8.20)
within 180 days of release *	171 (18.90)	26 (13.13)	145 (20.51)
**Outpatient encounter**			
within 30 days of release ***	91 (10.06)	5 (2.53)	86 (12.16)
within 180 days of release ***	222 (24.53)	29 (14.65)	193 (27.30)
2 or more outpatient encounters within 180 days of release **	155 (17.13)	19 (9.60)	136 (19.24)

M+: Medical code 2 and higher

M-: Medical code 1

P+: Psychological code 3 and higher

P-: Psychological code 2 and less

*: p < .05

**: p < .01

***: p < .001

Details from the multivariable models of outpatient and acute care utilization within 180 days of release are described in Table 3. The M + group was 1.65 (95% CI 1.12–2.44) times more likely to utilize outpatient services than the M- group. Outpatient utilization was positively associated with male sex, White race, and smoking. Although not associated with outpatient care utilization, the P + group was 1.98 (95% CI 1.36–2.88) times more likely to utilize acute care services than the P- group. Acute care utilization was positively associated with male sex and smoking. The M + group was not associated with acute care utilization.

**Table 3:** Multivariable analyses of predictors of healthcare utilization

	Odds ratios (+/- 95% CI)	
	Outpatient visit within 180d of release	Acute care visit within 180d of release
Age at release	1.013 (0.997–1.03)	1.013 (0.994–1.032)
Male vs. not male	1.805 (1.063–3.066) *	1.802 (1.027–3.162) *
Non-Hispanic vs. Hispanic	1.307 (0.936–1.825)	1.411 (0.975–2.04) †
White vs. non-White	2.039 (1.456–2.855) ***	1.293 (0.895–1.866)
Smoker vs. non-smoker	1.816 (1.293–2.55) ***	2.81 (1.928–4.096) ***
P + vs. P-	1.196 (0.851–1.68)	1.978 (1.36–2.879) ***
M + vs. M-	1.654 (1.12–2.441) **	0.904 (0.599–1.365)

M+: Medical code 2 and higher

M-: Medical code 1

P+: Psychological code 3 and higher

P-: Psychological code 2 and less

†: p < .10

*: p < .05

**: p < .01

***: p < .001

Hospitalization and mortality outcomes within 180 days of release were limited within the study population. Hospitalizations occurred for a total of 27 individuals and were more prevalent (n = 26, 3.7%) among individuals in the M + or P + group compared to the M- and P- group (n = 1, 0.5%) (Fisher exact p = .017). Fifteen total individuals died, and mortality prevalence was similar for those with and without a chronic condition (Fisher exact p = 1.00). Twelve deaths occurred within individuals designated M + or P+ (1.5%), and three deaths occurred in the M- and P- group (1.7%). The majority of deaths (n = 9, 60.0%) were categorized as accidental; 4 were categorized as homicide, 1 as suicide, and 1 of natural causes. Given the limited hospitalization and mortality outcomes, additional analyses were not conducted.

## Discussion

In this retrospective cohort study, we defined a continuum of care for people released from prison by assigning specific categories to the type and number of healthcare encounters. This novel model enabled an urban, safety-net healthcare institution to visualize cross-sectional, aggregate counts of people in stages along a continuum of accessing healthcare. A visual representation of this continuum reveals significant gaps in care which is concerning for a population with a high burden of chronic medical and psychological conditions.

Nearly 4 of every 5 individuals in our cohort were identified as having a chronic medical or psychological condition by CDOC. Despite a high prevalence of chronic health conditions, we saw low levels of accessing care. Notably, only 10% accessed an outpatient visit within 30 days of release, a period that carries an elevated risk for death (Binswanger et al., 2007, 2013; Fernandez L, 2022). Race, ethnicity, and gender were associated with healthcare utilization; those who were not White, Hispanic, and female were all less likely to have outpatient visits than their counterparts. Interventions aimed to improve access to care for people getting out of prison need to be sensitive to unique barriers faced by people of all races, ethnicities, and gender.

Our study demonstrated that treatment need categories assigned by a correctional system are associated with healthcare utilization in people released from prison. First, individuals with recognized chronic psychological conditions in prison were nearly twice as likely to have an acute care visit in the first 180 days after release, likely due to difficulty finding access to psychiatric specialists shortly after release or decompensated psychological conditions. The lack of an association between having a psychological condition and outpatient utilization after release unlikely reflects a lack of need for outpatient mental healthcare. Rather, because DH is not the largest safety net provider for mental health in Denver and people releasing from prison are often referred to providers approved by community corrections, this finding is not surprising. Second, individuals with recognized chronic medical conditions in prison were 1.7 times more likely to have an outpatient visit within 180 days of release. Despite barriers to care, many individuals with chronic medical conditions demonstrated their persistence in achieving access to outpatient health services. These findings indicate that correctional diagnoses can be used to tailor transitions of care programming to the sub-populations most likely to use health services after release. This is significant because limited resources are available for pre-release planning and reentry services.

This study was not powered to perform multivariable outcome analysis on hospitalization or death. It is, however, noteworthy that 12 of the 15 deaths occurred in individuals who had known chronic medical or psychological conditions and that drug overdoses, accidental poisonings, and injuries were the most common causes of death.

The findings in this study add to the literature by underscoring the importance of a comprehensive, population-based tool to evaluate access to care for people released from prison. A care continuum model allows us to assess the reach of our large, urban health system in its effort to provide timely, high-quality healthcare to this high-risk population. We use this information to develop interventions and collaborate with multiple stakeholders. Only by sharing data between correctional, community, and governmental stakeholders can we understand how, when, and where people released from prison access healthcare. A multi-institutional collaboration is necessary for continuity of care, improving public health, and maximizing the chance that those released from prison have the best opportunity possible to live healthy and productive lives. Existing programs that bridge care between these environments only serve a minute fraction of those releasing from prison. To improve population health, an ambitious, yet reasonable and necessary, goal is to promulgate this care continuum model in each US state. Taskforces with representation from state Medicaid programs, jail systems, state prisons, and healthcare organizations could be established and provided with funding to implement care continuums for people released from prison.

A variety of stakeholders could harness the data from this care continuum to design new interventions, target their limited resources, and evaluate the outcomes of these iterations. For example, a promising model of primary care, called the *transitions clinic model*, has been developed and replicated at dozens of community health centers across the US (Transitions Clinic Network, 2023). Using a community health worker with a personal history of incarceration, each clinic strives to improve access to care and health outcomes for people released from incarceration. Transitions clinics have been shown to improve engagement in primary care, reduce emergency department utilization, minimize future criminal justice system involvement, and achieve cost savings (Harvey et al., 2022; Wang et al., 2010, 2012, 2019). This model of primary care has yet to be scaled to match the numbers released from prison and remains targeted to a small percentage of the country, particularly those in an urban environment. A continuum of care for people released from prison would enable transition clinics across the country to evaluate their programs’ reach and to plan for targeted linkage efforts. Community supervision programs, healthcare clinics and hospitals, public health departments, community-based organizations that assist clients with reentry needs, and public payor sources (i.e., Medicaid and Medicare) would have various applications for this continuum of healthcare utilization since providing meaningful healthcare to people released from prison offers the potential to reduce recidivism, improve medical and psychological outcomes, prevent inappropriate and expensive emergency department utilization, and advance public health.

## Limitations

Our study has several important limitations. The cohort includes only individuals who were Medicaid-eligible and who signed a release of information allowing their information to be used to improve transitions of care; however, the release rosters capture 88% of those releasing from CDOC. We do not have data for people who are not covered by Medicaid or who declined to sign a waiver to improve care transitions. Our capacity to track healthcare utilization was limited to one healthcare institution, albeit one that provides more healthcare services to those covered by Medicaid than any other in the state. Lastly, data collection occurred approximately one year into the COVID-19 pandemic, which likely has multifactorial, unexpected effects on various factors such as the number of releases from prison, the number of people incarcerated, and influence on individual health risk behaviors and healthcare seeking behaviors among others.

## Conclusion

The continuum of care model can provide comprehensive, population-level data that illustrate where challenges exist on the path toward optimal health outcomes. Our application of the care continuum model revealed that people released from prison have robust challenges accessing any healthcare at all, have difficulty establishing and engaging in outpatient care in a timely manner, and experience additional barriers based on race, ethnicity, and gender. Our model will be used to target interventions that improve transitions of care from prison to DH. Great potential lies in (a) aggregating data from multiple institutions to create a global assessment of this population’s engagement with healthcare after release from prison and (b) in gathering all stakeholders together in an effort to improve access to care for this population that is at such high risk of death, overdose, recidivism, and other poor health outcomes.

### Electronic supplementary material

Below is the link to the electronic supplementary material.

Appendix

## Data Availability

Raw data for N = 905 individuals included in the final data can be found at https://github.com/DenverHealth-BH/Release_From_Incarceration_CC.
