# Supplementary material for: No health without access: using a retrospective cohort to model a care continuum for people released from prison at an urban, safety net health system
Source: Health Justice. 2023 Nov 18;11:49. doi: 10.1186/s40352-023-00248-3 (PMC10656837; doi:10.1186/s40352-023-00248-3)
Supplement: Supplementary file 1 — Appendix [file 40352_2023_248_MOESM1_ESM.docx]

APPENDIX

**DOC Clinical Services**

**Treatment Needs Guidelines**

Each treatment area within clinical services uses a five-point scale (1-5) to denote treatment needs levels.

**Psychological Needs Levels (P codes)**

P1 – No mental health treatment needs

· No historical, recent or current symptoms of any behavioral health problems;

· Mental health employees and contract workers provide counseling and treatment in the event of crises.

P2 – Low mental health treatment needs

· History of behavioral health problems or treatment in childhood, adolescence or adulthood that is not recent or current;

· Mental health employees and contract workers provide counseling and treatment in the event of crises.

P3 – Moderate mental health treatment needs

· All diagnoses of serious mental illness are a minimum of P3;

· Not all offenders at the P3 level take psychotropic medications, but all offenders taking psychotropic medications for mental health conditions are a minimum of P3.

P4 – High mental health treatment needs

· Psychiatric symptom severity is rated as high on the Brief Psychiatric Rating Scale (BPRS);

· Mental health resource consumption is rated as high on the Resource Consumption Scale (RCS);

· Behavioral health treatment and support is required for functioning in the correctional environment in general population. A change or decrease in the daily living skills of the P4 offender indicates the need for clinical intervention;

· Consultation with the mental health program administrator is required, and placement in Residential Treatment Program (RTP) may be needed.

P5 – Extreme mental health treatment needs

· Severe impairment due to psychiatric and behavioral health problems. These offenders are dangerous to themselves and/or others, and/or may be unable to perform tasks of daily living, such as hygiene and self-care;

· Conditions that are chronic and severe, that do not resolve quickly, and do not respond adequately to directions or education;

· Psychiatric symptom severity is rated as high on the BPRS;

· Mental health resource consumption is rated as high on the RCS;

· Functioning in the correctional environment requires a high level of mental health treatment, and psychiatric care, therefore, infirmary placement or RTP placement is required.

P Code Qualifiers: All P code levels of 3, 4, or 5 must have a qualifier. Qualifiers are indicative of the Diagnostic and Statistical Manual of Mental Disorders (DSM) diagnoses and are the primary focus of treatment. Qualifiers are to be consistent with the following definitions:

· M (Serious Mental Illness): This qualifier is used with a diagnosis of serious mental illness. These diagnoses will be considered primary and will determine the qualifier when other diagnoses are present. Examples include psychotic, bipolar, and major depressive disorders.

· O (Organicity): This qualifier is used when an offender meets the criteria for an organic mental disorder and may be coded with a specific DSM 5 organic mental disorder or neurocognitive disorders. These diagnoses have impacted cognitive functioning due to an organic process. Examples include traumatic brain injury and intellectual and developmental disabilities. Some diagnoses may be transient in nature and symptoms may resolve, or symptoms may persist.

· T (Temporary): This qualifier is used when an offender experiences stressors that are transient and temporary in nature. Short term treatment and support may be required for individuals to assist them through situational stress. Examples include various adjustment disorders, relational problems, and bereavement.

· N (Not otherwise specified): Not otherwise specified: This qualifier is used for all other diagnoses that do not meet the criteria for an M, O or T qualifier. Examples include anxiety and personality disorders.

· L (Low Frequency Monitoring): This qualifier is used when an offender is coded as a P3N or P3O. The offender is psychiatrically stable and functioning adequately in the facility.

**Medical Needs Levels (M Codes)**

M1: No medical needs

M2: Hematology, neurology, respiratory, ENT, GI, endocrine and cardiovascular diagnoses that can be managed by a primary care provider. Very early stages including HTN, diet controlled diabetes, IBS, etc.

M3: Hematology, neurology, respiratory, ENT, GI, endocrine and cardiovascular diagnoses that can be managed by a primary care provider.

M4: Hematology, neurology, respiratory, ENT, GI, endocrine and cardiovascular diagnoses requiring specialist care, any other diagnosis requiring ongoing specialty care.

M5: End stage chronic diseases, high medical needs patients in advanced stages of their progressive disease, terminal illness.
